# Haploinsufficiency of A20 in a Chinese child caused by loss-of-function mutations in TNFAIP3: A case report and review of the literature

**DOI:** 10.3389/fped.2022.990008

**Published:** 2023-01-11

**Authors:** Jing Liu, Yuese Lin, Xuandi Li, Hongjun Ba, Xiufang He, Huimin Peng, Shujuan Li, Ling Zhu

**Affiliations:** ^1^ Department of Pediatric Cardiology, The First Affiliated Hospital, Sun Yat-sen University, Guangzhou, China; ^2^ Key Laboratory on Assisted Circulation, Ministry of Health, Guangzhou, China

**Keywords:** A20, haploinsufficiency of A20, autoinflammatory disease, TNFAIP3, children

## Abstract

**Case Presentation:**

A 3-year-and-6-month-old child was reported to have recurrent high fever with generalized lymph node enlargement and significant elevation of inflammatory markers such as C-reactive protein and procalcitonin in tests. Later, whole exome sequencing determined that the child's disease was haploinsufficiency of A20 (HA20).

**Results:**

After immunosuppressive therapy, the child's symptoms improved significantly, and the inflammatory markers dropped to the normal range.

**Conclusion:**

Because of the characteristics of HA20, this disease is often underdiagnosed and misdiagnosed in clinical practice. By reporting this case of HA20 in a child, we hope to increase the awareness of this disease in the clinic.

## Introduction

Haploinsufficiency of A20 (HA20) is a rare monogenic autoinflammatory disease, which was first reported and named by Zhou et al. in 2016 (1). A20, also called TNFα-induced protein 3 (TNFAIP3), is a protein including 790 amino acids coded by the *TNFAIP3* gene, located on chromosome 6, which plays an important role in the negative regulation of inflammation and immunity. Loss of function mutation in *TNFAIP3* gene leads to HA20 and to an autoinflammatory or autoimmune disease. HA20 has a clinical presentation similar to infectious diseases and common rheumatic immune diseases, but with significant heterogeneity, and the correct diagnosis is often not determined in a timely manner in clinical practice (1, 2). Especially in the early stage of the disease, it often presents with recurrent high fever, significantly elevated inflammatory indexes, and ineffective anti-infective treatment. It is easy to misdiagnose or miss the diagnosis, and the possibility of HA20 needs to be considered. We present the clinical features of a case of HA20 in a child admitted to the Department of Pediatric Cardiology of the First Affiliated Hospital of Sun Yat-sen University, and conduct a literature review and discussion to improve the understanding of HA20 in clinical work.

**Table 1:** Laboratory examination results at admission.

Test items (reference values)	Results	Test items (reference values)	Results
Blood routine test		Autoimmune antibody	
White blood cell (4.00–10.00 × 10^9^/L)	9.98	ANA (0.00–12.00 U/ml)	33.3
Neutrophils (1.80–6.40 × 10^9^/L)	6.07	P-ANCA	Weakly positive
Hemoglobin (120–140 g/L)	104	C-ANCA	Negative
Platelet (100–300 × 10^9^/L)	371	ANCA-MPO	Negative
Acute-phase reactants		ANCA-PR3	Negative
PCT (0.00–0.05 ng/ml)	0.43	HLA-B27	Negative
C-reactive protein (0.00–10.00 mg/L)	75.50	SAA (0.00–6.40 mg/L)	675
ESR (0.00–20.00 mm/h)	25	RF (0.00–20.00 Ku/L)	12.30
Cytokine assay		Thyroid function	
IL-2 (0.00–5.71 pg/ml)	1.22	T3, T4, FT3, FT4	Normal
IL-4 (0.00–2.80 pg/ml)	3.34	TG-AB (≤3.99 IU/ml)	20.2
IL-6 (0.00–5.30 pg/ml)	15.35	TPO-Ab (≤9.00 IU/ml)	439.2
IL-17 (0.00–20.60 pg/ml)	14	Pathogenic examination	
TNF(0.00–4.60)	3.22	CMV-DNA (0–500 copies/ml)	Negative
IFN-*γ* (pg/ml)	4.61	Influenza virus antigen	Negative
Cellular immunization		EB-VCA-IgG (0.00–20.00 U/ml)	>750
CD19+ (7.0%–18.0%)	19.4	EB-VCA-IgM (0.00–40.00 U/ml)	12.70
CD3+ (620%–76.0%)	71.7	EB-VCA-IgA (0.00–10.00COI)	0.04
CD3+CD4+ (32.0%–46.0%)	34.5	EBV-DNA (0–500 Copies/ml)	Negative
CD3+CD8+ (18.0%–32.0%)	28.5	ASO (0.00–160.00 Ku/L)	228
NK cell (7.0%–18.0%)	8.3	Immunoglobulins	
Complements		IgE (0.00–120.00 IU/ml)	234.80
C3 (0.79–1.17 g/L)	1.28	IgA (1.45–3.45 g/L)	1.47
C4 (0.17–0.31 g/L)	0.28	IgG (10.13–15.13 g/L)	19.3
		IgM (0.92–2.04 g/L)	1.82

ANA, antinuclear antibodies; ESR, erythrocyte sedimentation rate; ANCA, anti-neutrophilic cytoplasmic antibodies; PR3, proteinase 3; MPO, myeloperoxidase; HLA-B27, human leukocyte antigen B27; SAA, serum amyloid A protein; RF, rheumatoid factor; TG-AB, thyroglobulin antibodies; TPO-Ab, thyroid peroxidase antibody; ASO, anti-streptolysin O; PCT, procalcitonin; IL, interleukin; TNF, tumor necrosis factor; IFN-γ, gamma-interferon. EB, Epstein-Barr; EBV, Epstein-Barr virus; VCA, viral capsid antigen; CMV, cytomegalovirus.

**Table 2:** Other examination results at admission.

Test items	Results
Pathogenetic examination	Epstein–Barr virus infection, herpes virus infection
Genetic testing for thalassemia	Alpha thalassemia gene SEA deletion (Southeast Asian type)
Bone marrow smear	Normal
Echocardiogram	Patent foramen ovale, mitral insufficiency (mild), aortic insufficiency (limited), tricuspid insufficiency (mild), low normal left ventricular systolic function
Abdominal ultrasound	Multiple lymph nodes in the peritoneal cavity and bilateral inguinal region
Biopsy of left inguinal lymph node	Reactive hyperplasia

## Case description

A male child, 3 years and 6 months old, was admitted to our hospital for fever and multiple skin rashes throughout the body. The child presented with toothache and fever of 38°C 10 days ago. The fever persisted even after the toothache improved. The patient was treated with antibiotics for 3 days at the outpatient department, but the fever did not improve, and the fever peaks at 40.3°C with 4–5 h intervals between fever peaks. The child was found to be hyperopic at about 6 months of age and is currently farsighted with 6.75 diopters. The father had a previous “transsphenoidal pituitary adenomectomy” for “non-functional pituitary macroadenoma, hypopituitarism.” The mother has “Mediterranean anemia.”

Physical examination at admission: lymphadenopathy was medium hard and movable in the neck, submandibular, and subchin bilaterally. Oral mucosa was smooth, pharynx was congested, tonsils were swollen (III°), and dental cavities were present. The spleen was palpable 1 cm below the ribs, medium in quality, and without percussion pain. The rest of the cardiopulmonary, abdominal, joint, and neurological examination showed no significant abnormality. The initial findings of the tests carried out after the child's admission to hospital are shown in Table 1 and Table 2.

The child was admitted to the hospital and given piperacillin sodium and tazobactam sodium for anti-infection due to the patient's infection indicators were well above normal. Seven days after the fever subsided, recurrent high fever with lethargy, oral and pendulous ulcers, generalized red maculopapular rash, and generalized superficial lymph node enlargement, liver, and spleen enlargement were seen again. The repeated blood test showed that C-reactive protein (CRP) was 108.66 mg/L, white blood cell count (WBC) was 9.56 × 10^9^/L, neutrophil numerals (NEUT#) was 8.54 × 10^9^/L, procalcitonin (PCT) was 127.00 ng/ml, lumbar puncture intracranial pressure was 155 mmH_2_O, and cerebrospinal fluid tests were negative. The fever subsided after treatment with acyclovir, meropenem, fluconazole, and gamma globulin 2 g/kg, but later there was still recurrent low-grade fever and peeling of skin at the junction of perianal, finger and toe nails, recurrent oral ulcers, and repeatedly increased CRP. The child's symptoms and medical history suggested that there might be an acute immune response to connective tissue disease or viral infection; then, we perfected whole exome sequencing. In the meantime, due to the high likelihood of connective tissue disease, he was given prednisone treatment (1 mg/kg qd) and his recurrent fever improved, but he still had recurrent oral ulcers. Then, the prednisone dose was gradually reduced. Based on the results for the full exome, methotrexate (5 mg qw) and thalidomide (12.5 mg qd) were added, after which the child did not have any more oral ulcers. Then, the child is discharged from the hospital. After 1 month of treatment at home, he returned to the hospital for follow-up: CRP was 0.94 mg/L, WBC was 6.14 × 10^9^/L, NEUT# was 3.85 × 10^9^/L, hemoglobin was 102 g/L, and erythrocyte sedimentation rate (ESR) was 40 mm/h; abdominal ultrasound showed multiple lymph nodes in the abdominal cavity and mesentery, smaller than before. Ten months later, we saw this child in our clinic and found that the patient did not develop recurrent fever and oral ulcers, and the blood test showed that CRP and PCT were reduced to normal, ESR fluctuated between 20 and 60 mm/h, and P-ANCA was still weakly positive.

**Figure 1:**
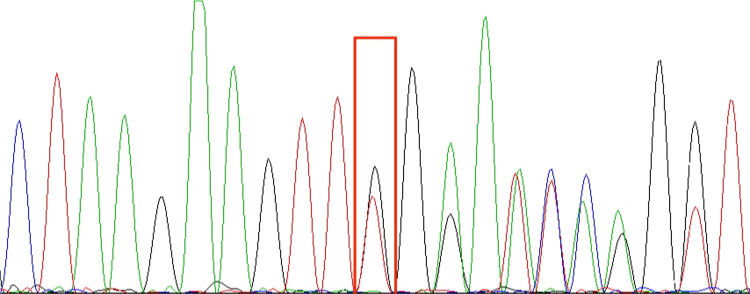
TNFAIP3 (chr6:138197151): NM_006290.3:c.653del:p.Leu218TrpfsTer10 sequencing results; red boxes are detection sites. In the picture, from top to bottom, are the first witness (**A**), the father of the first witness (**B**), and the mother of the first witness (**C**). The genetic test results indicate that the *TNFAIP3* gene variant in this pediatric patient is from the father and is a heterozygous mutation.

Whole exome sequencing revealed a heterozygous mutation in the affected *TNFAIP3* gene, chr6: 138197151, NM_006290.3 c.653del p.Leu218TrpfsTer10, derived from the father, consistent with HA20. This child's grandparents tested negative for the gene, suggesting that the mutation came from the father. The shift variant is a 1-base pair deletion, resulting in an out-of-frame transcription product and an early termination codon that may cause protein truncation or activation of nonsense-mediated mRNA degradation, resulting in loss of function of the protein product of the *TNFAIP3* gene. The downstream truncation variant of this variant is known to be pathogenic. The results are shown in the Figure 1.

## Literature review

To understand patients with HA20 onset and diagnosis in childhood, we searched the PubMed database using the keywords “HA20” from the year of creation to June 2022 and limited the search language to English, and exclude patients with onset or diagnosis in adulthood and young adulthood by carefully reading.

A total of 15 pediatric cases have been reported (Table 3). We further analyzed the data of these pediatric patients, including gender, age, and various common symptoms. Of these cases, six (40%) were male and nine (60%) were female. The number and percentage of these pediatric patients with various symptoms were as follows: nine (60%) had recurrent fever, nine (60%) had recurrent oral ulcers, four (26.6%) had recurrent genital ulcers, three (20%) had rashes, and six (40%) had arthritis. Most of these patients had different mutation sites in the *TNFAIP3* gene, with a small number of patients having mutations at the same site. In terms of treatment, pharmacological treatment in these patients also varied. The commonly used drugs were colchicine, prednisolone, sirolimus, tacrolimus, and infliximab.

**Table 3:** Cases of pediatric patients with HA20 reported in the literature.

Patients	Sex	Age (y)	TNFAIP3 Amino acid alteration	Recurrent fever	Recurrent oral ulcers	Recurrent genital ulcers	Rash	Arthritis	GI ulcers	Others	Treatment
1 (2)	M	9	p. Arg271*	Y	Y	Y	N	N	Y	Pericardial effusion	Colchicine
2 (2)	M	8	p. Gln338*	N	N	N	Y	N	Y	Pulmonary embolism, small vessel CNS vasculitis, inflammatory fibroepithelial polyp on the arytenoid	Without elaborating
3 (3)	F	14	p. Val489Alafs*7	N	N	N	N	N	Y	Insulin-dependent diabetes, cytopenias, hepatitis, enteropathy, interstitial lung disease	Prednisolone, sirolimus, tacrolimus, infliximab or rituximab, hematopoietic stem cell transplantation
4 (4)	M	4	p. His636Terfs*1	Y	Y	N	Y	N	N	—	Tocilizumab
5 (5)	M	6	p. Glu332*	Y	Y	N	Y	N	N	Vomit	Colchicine
6 (6)	F	8	p. Lys91*	N	N	N	N	Y	N	—	—
7 (7)	F	14	p. Gln187*	Y	N	N	N	N	N	Hypothyroidism, hepatic fibrosis	Etanercept, hydroxychloroquine, prednisolone, mycophenolate mofetil
8 (7)	M	5.1	p. Gln187*	Y	N	N	N	Y	Y	—	Etanercept, etiasa, methotrexate
9 (7)	F	9	p. Arg 87*	Y	Y	N	N	Y	N	—	Etanercept, prednisolone
10 (8)	M	7	p. Cys478*	Y	Y	Y	N	N	N	Pharyngalgia lymphadenopathy	Prednisolone, colchicine
11 (9)	F	4.6	p. Lys91*	N	N	N	N	Y	N	Autoimmune thyroid disorder, growth delay, cow's milk allergy	Thyroid replacement therapy, golimumab, azathioprine
12 (10)	M	9	Deletion of exons 2–3	Y	Y	Y	N	N	Y	−	Adalimumab, methotrexate
13 (11)	F	5	p.N98Tfs*25	N	Y	N	N	Y	N	−	Hydroxychloroquine, mycophenolate mofetil, IL-1 blocking therapy, azathioprine
14 (11)	M	2	p. R271X	N	Y	Y	N	N	N	—	Without elaborating
15 (12)	M	13	p. Met476Ile	Y	Y	N	N	Y	Y	Epstein–Barr virus infection, lymphadenopathy	Acyclovir, prednisolone

F, female; M, male; Y, yes; N, no; GI, gastrointestinal; age, age at diagnosis.

## Discussion

The young pediatric patient presented with recurrent high fever at the onset of the disease, with a generalized rash and recurrent oral ulcers, significantly elevated CRP (up to 108.66 mg/L), PCT (up to 127 ng/ml), and other infection indicators, no special abnormalities in rheumatologic immune indicators on comprehensive examination, and no improvement after anti-infection treatment, which was finally diagnosed as HA20 after whole exome sequencing. Due to HA20 being difficult to differentiate from other diseases, it is often initially diagnosed as other connective tissue diseases such as juvenile idiopathic arthritis, systemic lupus erythematosus (SLE), Behcet's disease, and rheumatoid arthritis. In particular, HA20 is different in that it does not have specific abnormalities in rheumatologic immune indexes (13).

In previous studies, elevated procalcitonin has been considered an important marker of bacterial infection in newborns and children, especially in febrile children without symptoms of local infection, which is why we made the diagnosis of infectious disease and administered antibiotics in this study (14). In healthy populations, circulating levels of PCT are usually below 0.1 ng/ml. In viral infections and inflammatory responses, PCT concentrations can increase to 1.5 ng/ml. In bacterial infections, PCT levels greater than 0.5 ng/ml are generally considered positive, and the highest levels may be >100,000 times the normal level. The highest value of PCT in this child was 127 ng/ml, which is well above the diagnostic range. Moreover, if the elevation of procalcitonin is due to bacterial infection, it will decrease rapidly after targeted antibiotic treatment; as described previously, the children in this study did not have a significant decrease in procalcitonin levels after antibiotic treatment (15). This suggests that in clinical practice, when faced with children with recurrent hyperthermia and elevated inflammatory markers (e.g., PCT), we need to consider not only infectious diseases and common rheumatic immune diseases such as juvenile idiopathic arthritis and systemic lupus erythematosus, but also the possibility of rare connective tissue diseases such as HA20 and genetic testing to help confirm the diagnosis if necessary.

HA20 is a monogenic genetic disorder whose pathogenesis is mainly associated with a TNFAIP3 minus function mutation affecting A20, which is encoded by the *TNFAIP3* gene, located on chromosome 6q23.3, and is a negative regulator of the tumor necrosis factor (TNF)-nuclear factor-κB signaling channel (16). A20 has an ovarian tumor region (OTU) structural domain in the N-terminal region, and a seven zinc finger (ZnF) structural domain in the C-terminal region. These two structural domains are negatively regulated by modification of the target proteins in the pathway. When the *TNFAIP3* gene is mutated, A20 expression is reduced, the negative regulation of the TNF-nuclear factor-κB signaling channel is weakened, and NK-*κ*B activation is increased, which in turn causes an increase in systemic inflammatory factors such as interleukin (IL)-1, IL-6, IL-8, and TNF-α, and patients develop a systemic inflammatory response (17). In our case, IL-6, IL-4, and IL-10 all showed elevations, which is similar to previous studies. No pure mutations have been reported in HA20, and the mutation in this disease is usually an autosomal dominant heterozygous mutation. Mutations in the *TNFAIP3* gene are widely distributed from the OTU to ZnF7 structural domains, with the OTU region and ZnF4 region being the most common, with a variety of mutations, including shift mutations, nonsense mutations, missense mutations, splice site mutations, and large deletions. The first two of which are more common in clinical practice (18). The patient in this case showed a shift mutation: TNFAIP3 (chr6:138197151): NM_006290.3:c.653del:p.Leu218TrpfsTer10, and this mutation has not been reported before.

The clinical symptoms of HA20 are variable, including recurrent fever, recurrent oral and genital ulcers, rash, arthritis, and gastrointestinal (GI) symptoms. A small number of patients also develop neurological symptoms and cardiovascular lesions. Significantly different from Behçet’s disease, HA20 patients experience a significant increase in inflammatory factors, especially during relapses, along with low titers of autoantibodies. In contrast, HA20 patients often have a combination of autoimmune diseases, such as SLE and autoimmune thyroid disease (18). Some patients have a probability of having abnormalities in relevant tests even though they do not meet the diagnostic criteria for these immunological disorders. For example, the patient in the report had fluctuating presence of various autoantibodies such as antinuclear antibodies and anti-dsDNA (2). Similar to previous reports, this patient in our case presented with abnormalities in the indicators, including antinuclear antibodies, serum amyloid A protein, and anti-neutrophilic cytoplasmic antibodies. There was also an abnormality in the thyroid antibody test, which may be related to the patient's abnormal immune function. Non-autoimmune complications such as IgA vasculitis, chronic hepatitis, nephrotic syndrome, and Hodgkin's lymphoma also occur in the HA20 patient population. A small number of patients have been reported HA20 co-infection with Epstein–Barr virus (12, 19) that is similar to the child in this case report, but whether Epstein–Barr virus infection is associated with HA20 requires further study. Patients with HA20 also present with some immunodeficiency symptoms such as recurrent infections, hypogammaglobulinemia, low IgA and IgE, low IgG, and CD19 and CD4 cytopenias (19–21). In these respects, this patient in the study showed high IgE and IgA on admission and an increase in CD19 cells on examination, but showed a decrease in IgA on retesting 2 months later, which differs from previous reports. HA20 has an earlier age of onset in childhood, and most present with recurrent fever and significant elevation of inflammatory factors, more severe GI symptoms, and more common autoimmune disease (18). In addition, the patient in our case presented with an elevated anti-streptolysin O, which was not submitted in the previous report. Whether this abnormality is related to HA20 requires further study.

The diagnosis of HA20 currently relies on whole exome sequencing. When patients present with recurrent fever, mouth ulcers, and genital ulcers, especially in the pediatric population, the possibility of HA20 needs to be considered. In the early stages of the disease, HA20 presents as recurrent mucosal ulcers and is therefore difficult to distinguish from diseases that also present as mucosal ulcers, such as Behcet's disease, rheumatoid arthritis, juvenile idiopathic arthritis, periodic fever with aphthous pharyngitis and adenitis, hyperimmunoglobulinemia D syndrome (HIDS), and inflammatory bowel disease (22). Whole exome sequencing of the ulcer helps clinicians to confirm the presence of the TNFAIP3 mutation and thus the diagnosis of HA20.

There is no standard treatment protocol for HA20, and commonly used drugs include systemic glucocorticoids, immunosuppressive agents, and biologic agents. Most patients respond well to treatment with systemic glucocorticoids. Colchicine is often used clinically in the treatment of HA20. Other immunosuppressive agents include methotrexate, cyclosporine, thalidomide, sirolimus, and tacrolimus. Biologic agents include anti-TNF-α drugs, anti-IL-1 drugs, anti-IL-6 drugs, and JAK inhibitors (13, 18, 21). A few patients were in remission from HA20 hematopoietic stem cell transplantation, both autologous and allogeneic (3, 22). However, it has been reported that a child who underwent autologous stem cell transplantation relapsed after 18 months of remission and had to be re-treated with immunosuppressive drugs (9). Some patients with recurrent respiratory infections underwent tonsillectomy. In this study, the patient showed significant improvement in fever symptoms after taking prednisone, but the oral ulcers were not controlled. After further treatment with methotrexate and thalidomide, oral ulcers improved, and the infection indexes were reduced to normal on review, and after 10 months of follow-up and examination, the dose of prednisone was gradually being reduced and the patient's condition did not recur.

In the face of patients with recurrent high fever with markedly elevated inflammatory factors, especially in younger pediatric patients like the one in this case, it is currently a challenge whether we can identify and diagnose HA20 in the early stages of its onset. Regarding the treatment of HA20, although the current multiple drug treatments and others can lead to remission, there is a lack of long-term follow-up data, and the long-term survival of patients needs further study.

## Data Availability

The original contributions presented in the study are included in the article/Supplementary Material, further inquiries can be directed to the corresponding author.
